# Early postoperative resistance index can predict radiocephalic arteriovenous fistula failure

**DOI:** 10.1177/11297298241295267

**Published:** 2024-11-01

**Authors:** Koji Inagaki, Chikao Onogi, Keita Iimuro, Akira Suzuki, Shin Furusawa, Masashi Tsuji, Toshiyuki Akahori

**Affiliations:** 1 Department of Nephrology, Chutoen General Medical Center, Shizuoka, Japan; 2 Department of Nephrology, Nagoya University, Graduate School of Medicine, Nagoya, Japan

**Keywords:** Resistance index, flow volume, radiocephalic, arteriovenous fistula, early, postoperative, ultrasonography

## Abstract

**Background:**

Ultrasonography is valuable for assessing arteriovenous fistula (AVF) maturation. Brachial artery flow volume (FV) measured at 6 weeks post-AVF creation can predict AVF failure. However, the association between early postoperative FV and AVF failure remains unclear. The resistance index (RI) may also serve as a prognostic factor for AVF maturation; however, it has not been extensively studied. Therefore, we aimed to investigate the relationship between AVF failure and early postoperative FV and RI.

**Methods:**

We retrospectively analyzed data from 102 patients (mean age, 67.8 ± 14.2 years; male, 68.6%; diabetes mellitus, 52.0%) who underwent new radiocephalic-AVF creation between January 2019 and December 2023 in Japan. An ultrasound device was used to measure brachial artery FV and RI on postoperative days 0 and 1. AVF failure was defined as occlusion or stenosis requiring vascular access intervention or surgical revision before the first cannulation, or cannulation difficulty or FV dysfunction requiring vascular access intervention or surgery at the first cannulation.

**Results:**

On postoperative day 0, FV was 383.1 ± 146.8 mL/min, and RI was 0.65 ± 0.11. On postoperative day 1, FV was 466.9 ± 179.2 mL/min, and RI was 0.62 ± 0.11. FV significantly increased, and RI was significantly reduced on postoperative day 1 compared to those on day 0 (*p* < 0.01). Multivariate analyses revealed that higher RI (per 0.1; odds ratio (OR), 2.16; 95% confidence interval (CI), 1.22–3.82), but not FV, was significantly associated with AVF failure on postoperative day 0. On postoperative day 1, lower FV (per 100 mL/min; OR, 0.63; 95% CI, 0.42–0.95) and higher RI (per 0.1; OR, 2.17; 95% CI, 1.16–4.05) were significantly associated with AVF failure.

**Conclusions:**

This study highlights RI as a predictor of AVF failure in the early postoperative period when vasospasm is likely to occur.

## Introduction

The number of patients with end-stage kidney disease is rising annually on a global scale.^1^ In Japan, the number of patients on dialysis is similarly increasing, reaching 347,671 patients by the end of 2020.^2^ Vascular access is essential for patients requiring maintenance hemodialysis (HD), with 89.7% using arteriovenous fistulas (AVFs).^3^ Ultrasonography is valuable for assessing AVF maturation, with brachial artery flow volume (FV) at 6 weeks postoperatively serving as a predictor of AVF maturation.^4,5^

The relationship between FV immediately after AVF creation and subsequent AVF maturity remains controversial. Zhu et al. and Shintaku et al. reported that FV on postoperative day 0 served as a predictor of AVF failure.^6,7^ Conversely, Ladenheim et al. demonstrated that intraoperative FV could not predict AVF maturation.^8^ Shimizu et al. reported that at 1 week postoperatively, the resistance index (RI), but not FV, predicted AVF failure.^9^ However, few studies have examined the relationships between FV, RI, and AVF maturity on the day after surgery (postoperative day 1). Despite extensive research on AVF success predictors over the years, several established predictors still leave room for improvement. Most studies focus on pre-operative factors, which are less predictive as they do not consider intraoperative variables. Therefore, in this study, we aimed to investigate the relationships between AVF failure and FV and RI on postoperative days 0 and 1.

## Methods

### Patients and methods

This study was a retrospective cohort study conducted at a single center in Japan. The study was approved by the ethics committee of Chutoen General Medical Center (Shizuoka, Japan; approval number KENI 152) and conducted in accordance with the ethical principles outlined in the Declaration of Helsinki. Written consent for data use was obtained from contactable patients whenever possible. Additionally, we made information about the study publicly available on our website, allowing patients the opportunity to opt-out.

Between January 2019 and December 2023, 129 patients underwent AVF surgery under local anesthesia, and during the perioperative period, we evaluated them using ultrasonography. Of these, eight and 15 patients underwent brachiocephalic AVF surgery in the elbow and AVF revision surgery due to AVF occlusion or stenosis, respectively. The remaining 106 patients underwent new radiocephalic (RC) AVF surgery in the forearm. Among these 106 patients, those who had not started hemodialysis (*n* = 2), had died before starting hemodialysis (*n* = 1), and had not been adequately evaluated using ultrasonography (*n* = 1) were excluded. Finally, 102 patients were included in the analysis.

### Preoperative assessment

The arteries and veins of the forearm of patients were assessed by performing ultrasonography prior to RC-AVF surgery using the SONIMAGE MX1 ultrasound system (KONICA MINOLTA, Tokyo, Japan) with a high-frequency linear probe (L11-3, KONICA MINOLTA). A tourniquet was wrapped around the upper arm of patients to dilate the vein, and the diameter of the forearm cephalic vein at the planned surgical site was measured. The patency of the cephalic vein at the elbow was confirmed, ensuring the absence of occlusions. After removing the tourniquet, the diameter of the radial artery at the planned surgical site was measured. The patency of the ulnar artery was confirmed using the color Doppler method. The diameters and FV of the brachial arteries were also measured. The FV was automatically calculated by the ultrasound scanner using the following equation:

$$\begin{array}{l} Flow\;volume\;=\;mean\;velocity\;\left(cm/s\right)\;\\ \;\;\;\;\;\;\;\;\;\;\;\;\;\;\;\;\;\;\;\;\;\;\;\;\;\;\;\;\;\;\;\;\;\;\;\;\;\;\;\;\;\;\;x\;\pi /4\;x\;{\left(diameter\right)}^{2}x\;60.^{7} \end{array}$$

### Surgical technique

After injecting the surgical site with 1% lidocaine, a transverse incision of approximately 5 cm was made in the forearm, 2–6 cm central to the wrist joint. The radial artery and cephalic vein were dissected and clamped, and incisions of 7–9 mm were made in both vessels. Next, a side-to-side vascular anastomosis was performed using a 7-0 polytetrafluorethylene suture. The presence of thrill, bleeding, or stenosis was evaluated after continuous suturing. Finally, the distal end of the vein was ligated and transected. If the artery and vein were not close enough for a side-to-side anastomosis, an end-to-side anastomosis was performed instead.

### Postoperative care and assessment

The AVF was evaluated by ultrasonography 1–3 h after AVF creation and again on the postoperative day 1, and FV and RI of the brachial artery were measured. The ultrasonographic assessment was repeated 1–2 weeks post-AVF creation. Subsequently, any abnormalities in the physical findings of the AVF were evaluated using ultrasonography. If AVF occlusion or stenosis was detected, vascular access intervention or surgery was performed. During the initial cannulation of the dilated vein for hemodialysis, the AVF was assessed using ultrasonography. The RI was automatically calculated by the ultrasound scanner using the following equation:

$$\begin{array}{l} RI\;=\;\left(peak\;systolic\;velocity\;-\;end\;diastolic\;velocity\right)\\ \;\;\;\;\;\;\;\;\;/\;peak\;systolic\;velocity.^{7} \end{array}$$

### Outcome

AVF maturation was deﬁned as at least one successful use of the AVF with two needles and a dialysis machine blood pump speed of 200 mL/min during HD, without the need for vascular access intervention or surgical repair. Conversely, AVF failure was defined as occlusion or stenosis requiring vascular access intervention or surgery before the first AVF cannulation, or cannulation difficulties or FV dysfunction requiring vascular access intervention or surgery at the first AVF cannulation.^7,10^ Outcome was defined as the presence or absence of AVF failure.

### Statistical analyses

Kolmogorov–Smirnov test was used to assess the distribution of continuous variables and confirm normality. Normally distributed variables were expressed as the mean ± the standard deviation and were compared using Student’s *t*-test. Nonparametric variables were expressed as the median and interquartile range (IQR) and compared using the Mann–Whitney *U* test. Categorical variables were expressed as a number and proportion and were compared using Fisher’s exact test. Comparisons of continuous variables between paired groups were performed using a paired *t*-test. Pearson’s correlation coefficient was used to determine the relationship between the two ultrasound parameters on postoperative days 0 and 1. Logistic regression analyses were used to determine the relationship between ultrasound parameters on postoperative days 0 and 1 and clinical parameters. For multivariate analysis, factors with *p*-values <0.10 in univariate analysis, as well as brachial artery FV and RI on postoperative days 0 and 1, were included.^4^ The results are expressed as odds ratios (ORs) with 95% confidence intervals (CIs). The optimal cut off values of FV and RI on postoperative days 0 and 1 for predicting AVF failure were evaluated using receiver operating characteristic (ROC) curves.

All statistical analyses were performed using the EZR software (Saitama Medical Center, Jichi Medical University, Saitama, Japan), a graphical user interface for R statistics (R Foundation for Statistical Computing, Vienna, Austria).^11^

## Results

Demographic and clinical characteristics of the patients are summarized in Table 1. The mean age was 67.8 ± 14.2 years; 70 (68.6%) patients were male, and 53 (52.0%) presented with diabetes mellitus (DM).

**Table 1.** Baseline clinical characteristics in 102 patients with AVF creation.

Variables	All (*N* = 102)	Maturation (*N* = 84)	Failure (*N* = 18)	*p* Value
Age, years	67.8 ± 14.2	66.2 ± 14.4	75.4 ± 11.0	0.012
Sex (female/male)	32 (31.4)/70 (68.6)	27 (32.1)/57 (67.9)	5 (27.8)/13 (72.2)	0.79
DM	53 (52.0)	43 (51.2)	10 (55.6)	0.80
Antiplatelet drug	24 (23.5)	19 (22.6)	5 (27.8)	0.76
BMI, kg/m^2^	23.9 ± 4.53	24.1 ± 4.66	22.6 ± 3.73	0.20
HD using CVC	23 (22.6)	19 (22.6)	4 (22.2)	1.0
HD over 3 months	4 (3.9)	3 (3.6)	1 (5.6)	0.55
sBP, mmHg	167.0 ± 30.1	168.5 ± 28.1	159.7 ± 38.4	0.26

AVF: arteriovenous fistula; DM: diabetes mellitus; BMI: body mass index; HD: hemodialysis; CVC: central venous catheter; sBP: systolic blood pressure.

Values are presented as mean (±SD) and numbers (%).

The preoperative and postoperative ultrasound findings and operative details are presented in Table 2. On postoperative day 0, FV was 383.1 ± 146.8 mL/min, and RI was 0.65 ± 0.11. The brachial artery diameters on postoperative day 0 were significantly increased compared with the preoperative diameters (*p* < 0.01). On postoperative day 1, FV increased to 466.9 ± 179.2 mL/min, and RI decreased to 0.62 ± 0.11. Both the increase in FV and the decrease in RI from day 0 to day 1 were statistically significant (*p* < 0.01). However, no significant difference in the brachial artery diameter was observed between postoperative day 0 and day 1.

**Table 2.** Preoperative and postoperative ultrasound findings and intraoperative details in 102 patients with AVF creation.

Variables	All (*N* = 102)	Maturation (*N* = 84)	Failure (*N* = 18)	*p* Value
Preoperative ultrasound finding				
Radial artery (mm)	2.78 ± 0.53	2.79 ± 0.54	2.73 ± 0.51	0.67
Cephalic vein (mm)	3.05 ± 0.99	3.14 ± 1.04	2.66 ± 0.60	0.060
Brachial artery (mm)	4.58 ± 0.70	4.56 ± 0.67	4.69 ± 0.83	0.46
Brachial artery FV (mL/min)	83.5 ± 45.8	85.7 ± 46.5	73.3 ± 42.2	0.30
Intraoperative detail				
Distance from wrist joint to AVF anastomosis (cm)	3.0 [3.0–5.0]	3.0 [3.0–5.0]	3.0 [3.0–4.88]	0.75
Side-to-side anastomosis with distal cephalic vein ligation	89 (87.3%)	73 (86.9%)	16 (88.9%)	1.0
Anastomotic diameter (mm)	8.0 [8.0–9.0]	8.0 [8.0–9.0]	8.0 [8.0–8.0]	0.50
Postoperative day 0 ultrasound finding				
FV (mL/min)	383.1 ± 146.8	394.7 ± 141.8	329.0 ± 161.2	0.085
RI	0.65 ± 0.11	0.63 ± 0.11	0.73 ± 0.11	0.001
Brachial artery (mm)	4.83 ± 0.67	4.81 ± 0.66	4.93 ± 0.74	0.49
Postoperative day 1 ultrasound finding				
FV (mL/min)	466.9 ± 179.2	487.7 ± 181.2	369.7 ± 135.2	0.011
RI	0.62 ± 0.11	0.60 ± 0.11	0.69 ± 0.10	0.001
Brachial artery (mm)	4.84 ± 0.65	4.82 ± 0.64	4.91 ± 0.68	0.62
Postoperative 1–2 weeks ultrasound finding				
FV (mL/min)	527.6 ± 224.3	554.9 ± 211.9	402.0 ± 243.1	0.008
RI	0.58 ± 0.13	0.56 ± 0.11	0.69 ± 0.16	<0.001

AVF: arteriovenous fistula; FV: flow volume; RI: resistance index.

Values are presented as mean (±SD), median [IQR], and numbers (%).

**Figure 1.**
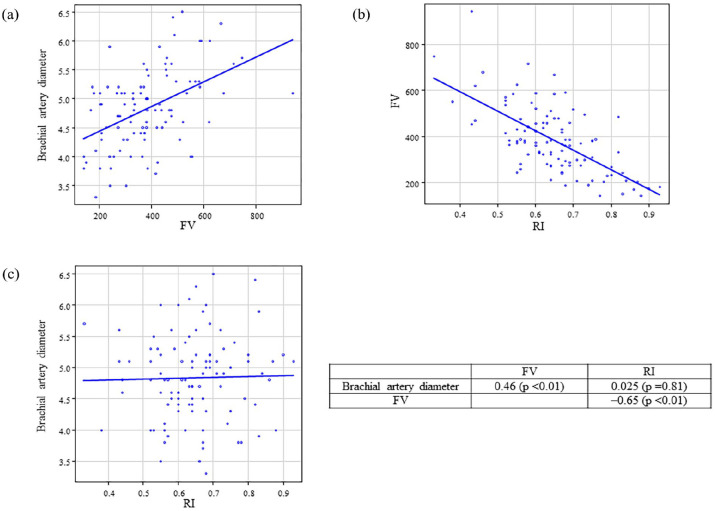
Correlation between brachial artery diameter, FV, and RI after AVF creation on the postoperative day 0. Correlation between (a) brachial artery diameter and FV, (b) FV and RI, and (c) brachial artery diameter and RI. FV: flow volume; RI: resistance index; AVF: arteriovenous fistula.

**Figure 2.**
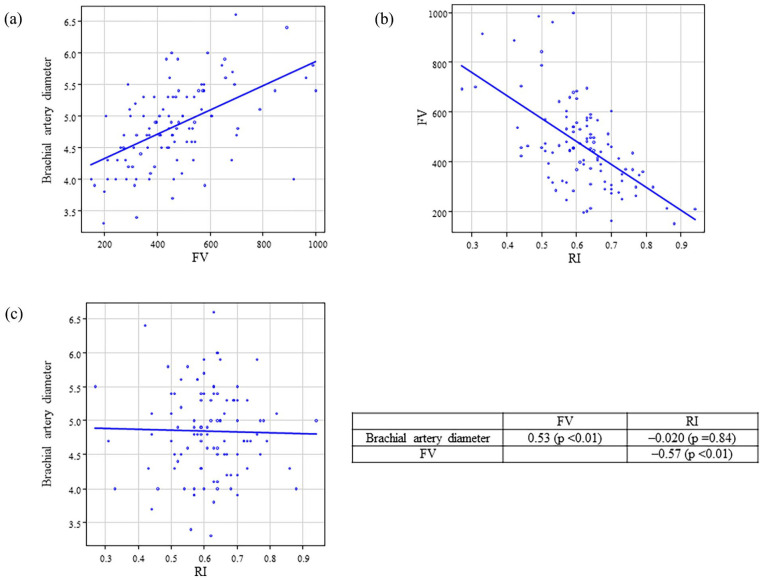
Correlation between brachial artery diameter, FV, and RI after AVF creation on the postoperative day 1. Correlation between (a) brachial artery diameter and FV, (b) FV and RI, and (c) brachial artery diameter and RI. FV: flow volume; RI: resistance index; AVF: arteriovenous fistula.

The correlations among brachial artery diameter, FV, and RI post-AVF creation are presented in Figures 1 and 2. FV was strongly correlated with brachial artery diameter on postoperative days 0 (*r* = 0.46, *p* < 0.01) and 1 (*r* = 0.53, *p* < 0.01). FV was also strongly correlated with RI on postoperative days 0 (*r* = −0.65, *p* < 0.01) and 1 (*r* = −0.57, *p* < 0.01). In contrast, the brachial artery diameter and RI were not significantly correlated.

The mean time from AVF creation to outcome was 81.2 ± 111.3 days. Eighty-four patients (82.4%) achieved unassisted AVF maturation, whereas 18 (17.6%) experienced AVF failure. Among those with AVF failure, four (22.2%) underwent repair surgery owing to AVF obstruction prior to initial AVF cannulation, nine (50.0%) required vascular access intervention owing to AVF stenosis or obstruction prior to initial AVF cannulation, and five (27.8%) underwent vascular access intervention owing to cannulation difficulty or AVF dysfunction at the first cannulation. The median time from AVF creation to first cannulation was 43.5 days (IQR, 15–112 days). Of the 23 patients on HD using central venous catheter (CVC), 15 (65.2%) were able to successfully undergo AVF cannulation within 2 weeks. Patients with AVF failure were significantly older than those with AVF maturation (Table 1). No significant differences were observed in sex, DM, or systolic blood pressure. Preoperative ultrasound findings showed a smaller vein diameter in the AVF failure group; however, this difference was not significant (Table 2). Intraoperatively, no significant differences were observed in the anastomosis method, diameter, or location between the AVF failure and maturation groups. On postoperative day 0, RI was significantly higher in the AVF failure group, although FV was not significantly different. In contrast, on postoperative day 1, the AVF failure group had a significantly higher RI and a significantly lower FV, compared with the maturation group (Table 2). Multivariate analysis, adjusted for age, preoperative cephalic vein diameter, and FV or RI, showed that a higher RI (per 0.1; OR, 2.16; 95% CI, 1.22–3.82) was significantly associated with AVF failure on postoperative day 0. In contrast, FV was not significantly associated with AVF failure on postoperative day 0 in either univariate or multivariate analyses. However, on postoperative day 1, lower FV (per 100 mL/min; OR, 0.63; 95% CI, 0.42–0.95) and higher RI (per 0.1; OR, 2.17; 95% CI, 1.16–4.05) were significantly associated with AVF failure (Table 3).

**Table 3.** Odd ratio of FV and RI on the postoperative days 0 and 1 by univariate and multivariate logistic regression analyses for AVF failure.

	Univariate analysis OR (95% CI)	Multivariate analysis OR (95% CI)
Postoperative day 0		
FV (per 100 mL/min)	0.70 [0.46–1.05]	0.73 [0.48–1.10]
RI (per 0.1)	2.30 [1.36–3.91]	2.16 [1.22–3.82]
Postoperative day 1		
FV (per 100 mL/min)	0.61 [0.41–0.90]	0.63 [0.42–0.95]
RI (per 0.1)	2.44 [1.37–4.35]	2.17 [1.16–4.05]

FV: flow volume; RI: resistance index; AVF: arteriovenous fistula; OR: odds ratio.

**Figure 3.**
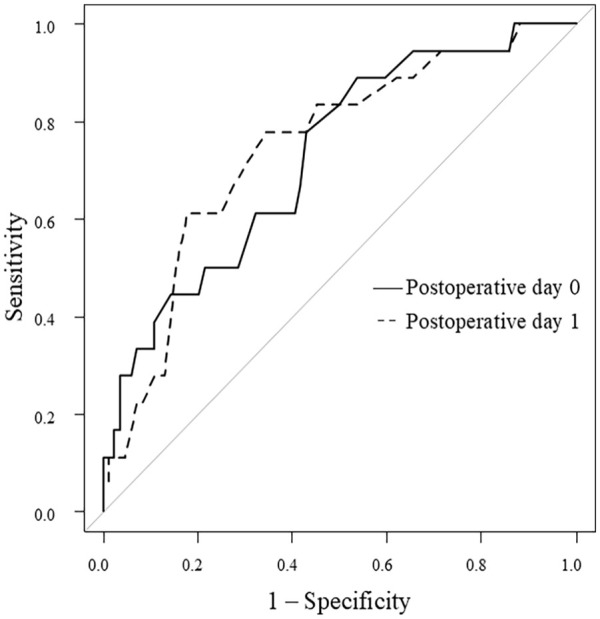
ROC curve for AVF failure of RI on the postoperative days 0 and 1. ROC: receiver operating curve; AVF: arteriovenous fistula; RI: resistance index.

The optimal cutoff values of FV and RI for predicting AVF failure were determined using ROC curve analysis. The areas under the ROC curves (AUC) for RI on postoperative days 0 and 1 were 0.73 and 0.75, respectively. On postoperative day 0, a cutoff value of 0.63 yielded a sensitivity of 0.889 and a specificity of 0.464. On postoperative day 1, a cutoff value of 0.64 yielded a sensitivity of 0.778 and a specificity of 0.665 for predicting AVF failure (Figure 3). The AUC value for FV on postoperative day 1 was 0.69. On postoperative day 1, a cutoff value of 320 mL/min yielded a sensitivity of 0.50 and a specificity of 0.833 for predicting AVF failure.

Patients with FV ⩾320 mL/min and <320 mL/min on the postoperative day 1 are shown in Table 4. Patients with FV <320 mL/min had significantly lower body mass index and smaller preoperative radial and brachial artery diameters, compared with those with FV ⩾320 mL/min. Patients with ⩾RI 0.64 and <0.64 on the postoperative day 1 are shown in Table 5. Patients with RI ⩾0.64 were significantly older and had a higher rate of side-to-side anastomosis with distal cephalic vein ligation, compared with those with RI <0.64. Among patients on HD using CVC, those with RI <0.64 had significantly more successful AVF cannulation within 2 weeks of AVF creation, compared with those with RI ⩾0.64 (Table 5).

**Table 4.** Comparison of patients with FV ⩾320 mL/min and <320 mL/min on the postoperative day 1.

Variables	FV ⩾320 mL/min (*N* = 79)	FV <320 mL/min (*N* = 23)	*p* Value
Clinical finding			
Age, years	67.4 ± 13.9	69.1 ± 15.5	0.63
Sex (female/male)	21 (26.6)/58 (73.4)	11 (47.8)/12 (52.2)	0.074
DM	43 (54.4)	10 (43.5)	0.48
Antiplatelet drug	20 (25.3)	4 (17.4)	0.58
BMI, kg/m^2^	24.6 ± 4.62	21.4 ± 3.22	0.003
HD using CVC	15 (19.0)	8 (34.8)	0.16
HD over 3 years	3 (3.8)	1 (4.3)	1.0
sBP, mmHg	167.7 ± 27.2	164.6 ± 39.3	0.67
Preoperative ultrasound finding			
Radial artery (mm)	2.85 ± 0.55	2.52 ± 0.39	0.008
Cephalic vein (mm)	3.15 ± 1.00	2.72 ± 0.89	0.068
Brachial artery (mm)	4.70 ± 0.69	4.17 ± 0.53	0.001
Brachial artery FV (mL/min)	90.3 ± 48.0	60.3 ± 27.1	0.005
Intraoperative detail			
Distance from wrist joint to AVF anastomosis (cm)	3.0 [3.0–4.8]	3.0 [3.0–5.0]	0.98
Side-to-side anastomosis with distal cephalic vein ligation	68 (86.1%)	21 (91.3%)	0.73
Anastomotic diameter (mm)	8.0 [8.0–9.0]	8.0 [8.0–9.0]	0.63
Postoperative clinical findings in patients on HD using CVC			
AVF cannulation success within 14 days	12 (80.0)	3 (37.5)	0.071

FV: flow volume; DM: diabetes mellitus; BMI: body mass index; HD: hemodialysis; CVC: central venous catheter; sBP: systolic blood pressure; AVF: arteriovenous fistula.

Values are presented as mean (±SD), median [IQR], and numbers (%).

**Table 5.** Comparison of patients with RI <0.64 and ⩾0.64 on the postoperative day 1.

Variables	RI <0.64 (*N* = 59)	RI ⩾0.64 (*N* = 43)	*p* Value
Clinical finding			
Age, years	64.1 ± 15.1	72.9 ± 11.2	0.002
Sex (female/male)	22 (37.3)/37 (62.7)	10 (23.3)/33 (76.7)	0.19
DM	29 (49.2)	24 (55.8)	0.55
Antiplatelet drug	11 (18.6)	13 (30.2)	0.24
BMI, kg/m^2^	24.4 ± 4.94	23.2 ± 3.85	0.19
HD using CVC	17 (28.8)	6 (14.0)	0.095
HD over 3 years	3 (5.1)	1 (2.3)	0.64
sBP, mmHg	164.8 ± 30.5	170.0 ± 29.7	0.40
Preoperative ultrasound finding			
Radial artery (mm)	2.81 ± 0.60	2.74 ± 0.42	0.54
Cephalic vein (mm)	3.16 ± 1.02	2.90 ± 0.93	0.18
Brachial artery (mm)	4.53 ± 0.71	4.65 ± 0.67	0.39
Brachial artery FV (mL/min)	84.7 ± 50.5	81.9 ± 39.1	0.76
Intraoperative detail			
Distance from wrist joint to AVF anastomosis (cm)	3.0 [3.0–5.0]	3.0 [3.0–4.0]	0.16
Side-to-side anastomosis with distal cephalic vein ligation	48 (81.4%)	41 (95.3%)	0.040
Anastomotic diameter (mm)	8.0 [8.0–9.0]	8.0 [8.0–9.0]	0.98
Postoperative clinical findings in patients on HD using CVC			
AVF cannulation success within 14 days	14 (82.4)	1 (16.7)	0.009

RI: resistance index; DM: diabetes mellitus; BMI: body mass index; HD: hemodialysis; CVC: central venous catheter; sBP: systolic blood pressure; FV: flow volume; AVF: arteriovenous fistula.

Values are presented as mean (±SD), median [IQR], and numbers (%).

## Discussion

In this study, we evaluated the association between ultrasound findings immediately after AVF creation and subsequent AVF maturation. FV on postoperative day 1, and not postoperative day 0, emerged as a significant predictor of AVF failure. In contrast, RI was a significant prognostic factor for AVF failure on both postoperative days 0 and 1. To the best of our knowledge, this is the first study to report ultrasound evaluation of AVF on two consecutive postoperative days for predicting AVF failure.

Several studies have evaluated the relationship between FV and AVF failure during the early postoperative period. Zhu et al. reported that an FV >529 mL/min on postoperative day 0 was associated with AVF maturation.^6^ Shintaku et al. reported that FV <235 mL/min on postoperative day 0 was associated with AVF failure.^7^ However, in our study, FV on postoperative day 0 was not associated with AVF failure, whereas FV on postoperative day 1 was. Given that AVF was created using local anesthesia and FV was measured within a few hours post-surgery, we hypothesize that this discrepancy is related to blood vessel spasms. Wong et al. reported that intraoperative FV was not associated with AVF maturation, likely owing to vasospasm of the blood vessels.^12^ Aitken et al. reported that AVF creation after brachial plexus block anesthesia, rather than local anesthesia, reduces vasoconstriction and improves AVF patency.^13^ In the present study, FV was higher on postoperative day 1, when it was less affected by vasospasm, than on postoperative day 0. Additionally, the brachial artery diameter was included in the FV calculation formula. Given that FV on postoperative day 0 was small, the size of the brachial artery had a marked influence.

Only a few studies have examined the relationship between postoperative RI and subsequent AVF maturation. Shintaku et al. reported that a brachial artery RI of ⩾0.63 on postoperative day 0 decreased AVF patency.^7^ Shimizu et al. reported that a brachial artery RI of ⩾0.65 on postoperative day 7 also decreased AVF patency,^9^ suggesting that RI reflects arterial structure and venous twist or flexion. Recently, Giannikouris et al. investigated brachial artery diameter, FV, and RI during the first 90 days after AVF creation.^14^ They demonstrated that brachial artery diameter and FV peaked on days 60 and 90, respectively, with no significant changes in day 7 values during the follow-up period. In contrast, RI reached a minimum on day 30, with no significant changes after day 2. Given that RI peaks within a few days after surgery and does not correlate with brachial artery diameter, the RI on postoperative day 0 can serve as an early predictor of AVF failure.

If postoperative RI is low, early AVF cannulation may have higher rates of success. In Japan, it is recommended that AVF cannulation be performed after 2 weeks of AVF creation.^15^ The Dialysis Outcomes and Practice Patterns Study reported a 2.1-fold increased risk of AVF failure for cannulation within 2 weeks of AVF creation.^16^ However, early cannulation can reduce the need for a temporary catheter and complications, such as infection.^17^ Recently, Shimizu et al. reported that cannulation within 14 days after AVF creation did not change the AVF patency rate.^9^ They also reported that early cannulation in patients with RI ⩾0.65 one week postoperatively significantly decreased the AVF patency rate. Consistent with this, in our study, the patients undergoing HD with CVC with RI <0.64 on the postoperative day 1 had significantly more successful AVF cannulation within 2 weeks of AVF creation. Therefore, measuring RI in the early postoperative period may predict the success or failure of AVF cannulation.

This study had several limitations. First, the small study population prevented complete adjustment for known prognostic factors. Second, given the retrospective observational nature of the study, we could not rule out any residual confounding factors. However, clinical records were continuously maintained in a clinical database, and there were few missing data. Third, the speed of the blood pump in the dialysis machine (200 mL/min), used to define clinical maturation, was low because the blood pump speed in the dialysis machine is lower in Japan than in other countries.^15^ Therefore, large-scale and prospective international studies are needed in the future.

In conclusion, this study found that on postoperative day 0 after RC-AVF creation, when vasospasm affects the AVF, RI, but not FV, serves as a predictor of RC-AVF failure. Assessing RI in the early postoperative period helps predict the likelihood of successful early cannulation of AVF.
